# Radiation shielding performance of metal oxides/EPDM rubber composites using Geant4 simulation and computational study

**DOI:** 10.1038/s41598-023-34615-9

**Published:** 2023-05-12

**Authors:** Mahmoud T. Alabsy, Mohamed Abd Elzaher

**Affiliations:** 1 Physics Department, Faculty of Science, Alexandria University, Alexandria, 21511 Egypt; 2 Department of Basic and Applied Science, Faculty of Engineering, Arab Academy for Science, Technology, P.O 1129, AL Alamien, Egypt

**Keywords:** Nanoscience and technology, Physics

## Abstract

This paper aimed to evaluate the shielding performance of ethylene propylene diene monomer (EPDM) rubber composites filled with 200 phr of different metal oxides (either Al_2_O_3_, CuO, CdO, Gd_2_O_3_, or Bi_2_O_3_) as protective materials against gamma and neutron radiations. For this purpose, different shielding parameters, including the linear attenuation coefficient (μ), mass attenuation coefficient (μ/ρ), mean free path (MFP), half value layer (HVL), and tenth value layer (TVL), were calculated in the energy range between 0.015 and 15 MeV by using the Geant4 Monte Carlo simulation toolkit. The simulated μ/ρ values were validated by the XCOM software to examine the precision of the simulated results. The maximum relative deviation between the Geant4 simulation and XCOM was not greater than 1.41%, confirming the accuracy of the simulated results. Based on μ/ρ values, other significant shielding parameters such as effective atomic number (Z_eff_), effective electron density (N_eff_), equivalent atomic number (Z_eq_), and exposure buildup factor (EBF) were also computed to explore the potential usage of the proposed metal oxide/EPDM rubber composites as radiation protective materials. The study demonstrates that the gamma-radiation shielding performance of the proposed metal oxide/EPDM rubber composites are increasing in the order of EPDM < Al_2_O_3_/EPDM < CuO/EPDM < CdO/EPDM < Gd_2_O_3_/EPDM < Bi_2_O_3_/EPDM. Furthermore, three sudden increases in the shielding capability in some composites occur at 0.0267 MeV for CdO/EPDM, 0.0502 MeV for Gd_2_O_3_/EPDM, and 0.0905 MeV for Bi_2_O_3_/EPDM composites. This increase in the shielding performance is due to the K absorption edges of Cd, Gd, and Bi, respectively. Regarding the neutron shielding performance, the macroscopic effective removal cross-section for fast neutrons (Ʃ_R_) was evaluated for the investigated composites using MRCsC software. The highest Ʃ_R_ is obtained for Al_2_O_3_/EPDM, while the lowest Ʃ_R_ is obtained for EPDM rubber with no metal oxide content. According to the obtained results, the investigated metal oxide/EPDM rubber composites can be employed as comfortable clothing and gloves designed for workers in radiation facilities.

## Introduction

Natural and artificial sources of ionizing radiation are broadly used in many applications such as medicine, industry, agriculture, space missions, nuclear power plants and scientific research. Despite the indispensable usages of ionizing radiation in various fields, it can also cause deterministic or stochastic effects on human health^1^. For this reason, it is essential to employ effective shielding materials to attenuate the ionizing radiation and protect the people and the environment from these harmful effects. To design an effective radiation shielding material, the interaction of the selected protective material with different types of radiations (e.g. neutrons, gamma rays, and charged particles) must be taken into account. For example, high energetic fast neutrons is slowed down by heavy elements through the process of inelastic scattering^2^. Intermediate energy neutrons can be moderated by elastic scattering with light elements such as hydrogen, while, thermal neutrons are absorbed by elements of high absorption cross section such as boron and cadmium^3^. On the other hand, γ-rays and X-rays can be attenuated by high dense elements with high atomic number such as lead, tungsten and bismuth. Therefore, polymer composites incorporate light and heavy elements used as multifunctional protective shielding materials against neutrons and gamma-rays have gained much interest in the last decade^4^.

Various polymers such as, high density polyethylene^5^, natural rubber^6^, epoxy resin^7^, polyester^8^, polyimide^9^, and polystyrene^10^, and Polyvinyl chloride^11^ were studied as radiation shielding matrixes against radiations. Ethylene-propylene-dine monomer (EPDM), which is a synthetic rubber often used in daily applications^12^, is also one of the polymer materials that can be used as a radiation-protective material in nuclear applications. Özdemir et al. demonstrated that EPDM rubber with addition of boron compounds could be used as effective and flexible neutron shielding composite^13,14^. In addition, composites based on EPDM rubber dispersed with PWO fillers for gamma radiation shielding applications were reported^15^. Moreover, EPDM rubber composites containing different metal oxides (Fe_3_O_4_,W_2_O_3_ or Bi_2_O_3_) were investigated as effective, flexible, lead-free, gamma-ray shielding materials to replace lead-containing materials^16^.

As indicated from the literature, several studies have been reported the gamma-radiation shielding properties of EPDM rubber composites in terms of linear attenuation coefficient (μ) and mass attenuation coefficient (μ/ρ). However, the review of the literature reveals that other important shielding parameters such as effective atomic number (Z_eff_), effective electron density (N_eff_), equivalent atomic number (Z_eq_) and exposure buildup factor have not been encountered for EPDM rubber composites. Monte Carlo simulation is an efficient tool that can be employed to examine the capability of a tested material to be used for γ-radiation shielding applications. For this purpose, several Monte Carlo simulation codes such as Geant4^17^, MCNP^18^, and FLUKA^19^ are used to simulate the passage of radiation through matter. Such simulation codes offer accurate and flexible use, compared to the experimental procedure, to predict the radiation shielding characteristics of any proposed material. Furthermore, the photon cross sections for elements, compounds and mixtures can also be determined theoretically from the NIST-XCOM database^20^ to check the accuracy of a conducted simulation.

This research aimed to investigate the shielding characteristics of EPDM rubber composites filled with different metal oxides (Al_2_O_3_, CuO, CdO, Gd_2_O_3_ and Bi_2_O_3_) to explore their potential usage as radiation protective materials. To this end, the linear attenuation coefficients (μ) of the suggested EPDM rubber composites were simulated using Geant4 simulation code. Based on the simulated μ values and the density of these composites, mass attenuation coefficient (μ/ρ), mean free path (MFP), half value layer (HVL), and tenth value layer (HVL) were determined. μ/ρ values were also compared to those acquired from the XCOM database to examine the precision of our simulated results. Moreover, other significant shielding parameters such effective atomic number (Z_eff_), effective electron density (N_eff_), equivalent atomic number (Z_eq_) and exposure buildup factor were also computed in the energy range between 0.015 and 15 MeV. Finally, the macroscopic effective removal cross section for fast neutrons (Ʃ_R_) was theoretically computed to assess the attenuation capability of the investigated metal oxide/EPDM rubber composites against fast neutrons.

## Materials and methods

### EPDM rubber composites

The proposed EPDM rubber in this investigation, obtained from Ref.^21^, consists of 52 wt% ethylene, 43.7 wt% propylene, and 4.3 wt% ethylidene norbornene in each 100 phr (parts per hundred of rubber) of EPDM rubber. The metal oxide/EPDM rubber samples are supposed to be composed of 100 phr of EPDM rubber and other compounding agents given in Table 1 with additions of 200 phr metal oxide fillers (either Al_2_O_3_, CuO, CdO, Gd_2_O_3_ or Bi_2_O_3_). The chemical formulas and the corresponding weight fractions for all the ingredients composing the metal oxide/EPDM rubber composites are listed in Table 1.

**Table 1:** Material formulations and the corresponding weight fractions of metal oxide/EPDM composites.

Ingredients	Chemical formulas	Content (phr)^a^	Weight fractions %
Ethylene	C_2_H_4_	52	13.36
Propylene	C_3_H_6_	43.7	11.23
Ethylidene norbornene	C_9_H_12_	4.3	1.1
Zinc oxide	ZnO	5	1.28
Stearic acid	C_18_H_36_O_2_	1	0.26
Sulfur	S	1.5	0.39
N-cyclohexyl-2-benzthiazylsulfenamide	C_13_H_16_N_2_S_2_	0.8	0.21
Ttetramethylthiuram disulfide (TMTD)	C_6_H_12_N_2_S_4_	1	0.26
Carbon black	C	40	10.27
Paraffinic oil	C_15_H_11_ClO_7_	40	10.27
Metal oxide	Either Al_2_O_3_, CuO, CdO, Gd_2_O_3_ or Bi_2_O_3_	200	51.37

^a^*phr* parts per hundred parts of rubber.

### Theoretical background

The attenuation of gamma radiation of certain energy through a target absorber can be described by the well-known Beer–Lambert’s law given by Eq. (1)^22^, from which the most important shielding parameter, the linear attenuation coefficient (μ), can be determined:

1 $$I={I}_{0 }{e}^{-\mu x}$$

where I and I_0_ are the transmitted and incident photon intensities, respectively, passing through an absorber of thickness *x*. In our Geant4 simulation code, I_0_ is the number of primary events incident on the composite and I is the number of events transmitted without any interaction with the material.

To examine the capability of the investigated EPDM composites as gamma-ray shielding materials, the mass attenuation coefficient (μ/ρ) was then computed by simply dividing the estimated linear attenuation coefficient (μ) of a given composite by its density (ρ). Theoretically, (μ/ρ) can also be evaluated by Eq. (2)^23^:

2 $$\frac{\mu }{\rho }=\sum_{i}{w}_{i}{\left(\frac{\mu }{\rho }\right)}_{i}$$

where (μ/ρ)_i_ and w_i_ are the mass attenuation coefficient and the weight fraction of the ith constituent element in the composite sample, respectively.

As the incident photons interacts with the EPDM sample, the average distance a photon travels between two successive interactions is known as the mean-free path (MFP) and is defined by Eq. (3)^24^:

3 $$MFP=\frac{1}{\mu }$$

The half-value layer (HVL) and tenth-value layer (TVL), are essential shielding parameters which must be taken account in choosing an appropriate radiation protective material. These parameters represent the attenuator thicknesses needed to diminish the incident gamma-ray intensity to 50% and 10% of its initial value and determined from Eqs. (4) and (5) respectively^25^:

4 $$HVL=\frac{\mathrm{ln}2}{\mu }$$

5 $$TVL=\frac{\mathrm{ln}10}{\mu }$$

The effective atomic number (Z_eff_) is a useful photon interaction parameter that depends on the photon energy and is used to characterize the shielding properties of composites in terms of pure elements. Z_eff_ values for the proposed metal oxides/EPDM rubber composites can be computed using Eq. (6)^26^:

6 $${Z}_{eff}=\frac{{\sum }_{i}{f}_{i}{A}_{i}{\left(\frac{\mu }{\rho }\right)}_{i}}{{\sum }_{j }{f}_{i }\frac{{A}_{j}}{{Z}_{j}}{\left(\frac{\mu }{\rho }\right)}_{j}}$$

where f_i_, A_i_, and Z_i_ are the molar fraction, the atomic weight, and the atomic number of the ith constituent element in the composite material.

The number of electrons per unit mass of the composite material is called the effective electron density (N_eff_), measured in electrons/g, and is calculated from Eq. (7)^27^:

7 $${N}_{eff}=\frac{{N}_{A}{Z}_{eff}}{\langle A\rangle }$$

where $\langle A\rangle ={\sum }_{i}{f}_{i}{A}_{i}$ is the average atomic mass of the composite material and N_A_ is the Avogadro’s number.

To develop an effective shielding material, the exposure-buildup factor (EBF) must be taken into account to correct the attenuation calculations due to buildup of secondary photons resulted from Compton scattering. To compute the EBF for the proposed metal oxide/EPDM rubber composites, Geometric-Progression fitting method (GP) was utilized.

The calculations of the EBF were done according to the following steps:

- Equivalent-atomic number (Z_eq_), which is an energy dependent parameter relating the properties of the investigated metal oxide/EPDM rubber composites in terms of its equivalent elements, was first determined using the following formula^17^:
  8 $${Z}_{eq}=\frac{{Z}_{1}\left(\mathrm{log}{R}_{2}-\mathrm{log}R\right)+{Z}_{2}\left(\mathrm{log}R-\mathrm{log}{R}_{1}\right) }{\mathrm{log}{R}_{2}-\mathrm{log}{R}_{1}}$$
  where R_1_ and R_2_ are the (μ_Comp_/μ_total_) ratios corresponding to the elements with atomic numbers Z_1_ and Z_2_ respectively, and R is the (μ_Comp_/μ_total_) ratio for the polymer selected at a specific energy, which lies between ratios R_1_ and R_2_.
- The computed Z_eq_ values of the investigated polymers were then used to interpolate GP fitting exposure buildup factor coefficients (b, c, a, X_K_, d) in the energy range 0.015–15 MeV using the interpolation formula^28^(9):
  9 $$C=\frac{{C}_{1}\left(\mathrm{log}{Z}_{2}-\mathrm{log}{Z}_{eq}\right)+{C}_{2}\left(\mathrm{log}{Z}_{eq}-\mathrm{log}{Z}_{1}\right) }{\mathrm{log}{Z}_{2}-\mathrm{log}{Z}_{1}}$$
  where C_1_ and C_2_ are GP fitting parameters, acquired from ANSI/ANS-6.4.3 standard database^28^, corresponding to Z_1_ and Z_2_ between which Z_eq_ of the selected composite lies.
- As a final step, the EBF for the studied metal oxides/EPDM rubber composites were then calculated by means of the estimated GP fitting parameters, using the following equations^29,30^:
  10 $$B\left(E,x\right)=1+\frac{b-1}{K-1} \left({K}^{x}-1\right) , K\ne 1$$
  and
  11 $$B\left(E,x\right)=1+\left(b-1\right)x , K=1$$
  where
  12 $$K\left(E,x\right)=c{x}^{a}+d\frac{\mathrm{tanh}\left(x/{X}_{K}-2\right)-\mathrm{tanh}\left(-2\right)}{1-\mathrm{tanh}\left(-2\right)} \mathrm{for x }\le 40\mathrm{ mfp}$$
  where E is incident photon energy and x is the mfp.

### Geant4 simulation

Based on Monte Carlo methods and C++ programming language, Geant4 is an object-oriented toolkit for the simulation of the passage of different kinds of particles through matter^31^. Geant4 is utilized by a broad variety of user communities in different application disciplines such as high energy physics, calorimetry studies, space science, radiotherapy and radiation protection. The present study is an attempt to employ the Geant4 simulation toolkit to measure the linear attenuation coefficients for the chosen metal oxides/EPDM rubber composites at a wide energy range varied between 0.015 and 15 MeV. The simulation was implemented in a Linux operating system employing Geant4 version 10.7.

**Figure 1:**
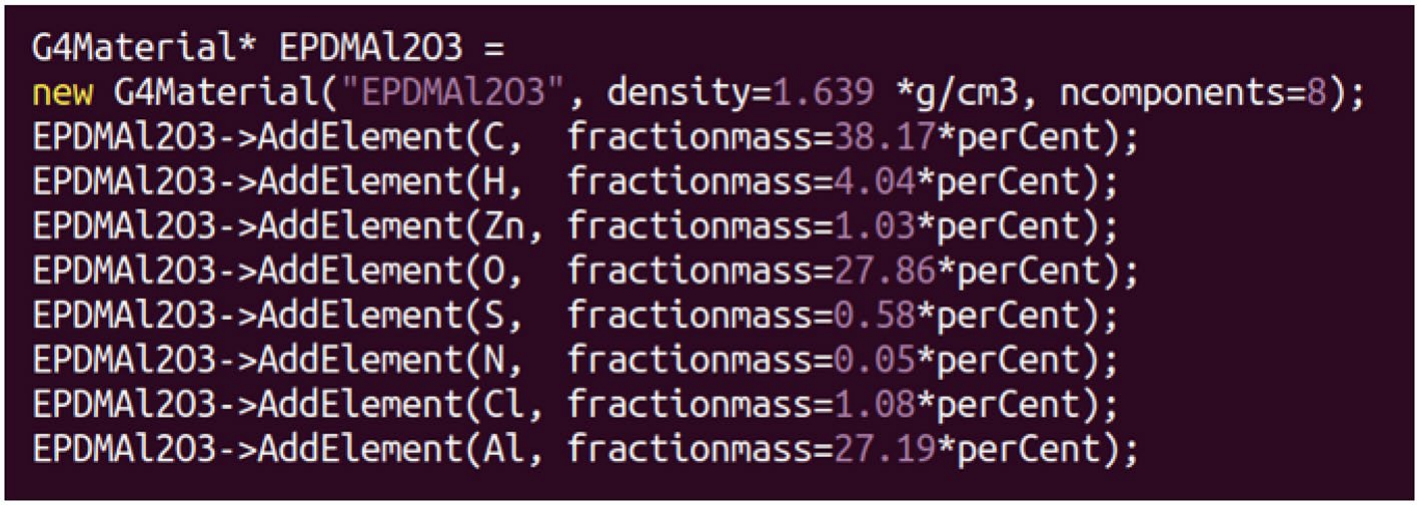
Geant4 code used to build the Al_2_O_3_/EPDM rubber composite using the G4Material class.

To conduct such a simulation code, three main Geant4 classes were implemented: DetectorConstruction, PhysicsList, and ActionInitialization. The DetectorConstruction class constructs the detector geometry and defines its materials. The setup consists of a single cubic box of side 10 cm of a homogeneous material. To build a selected composite, firstly, the G4Element class is used to define the properties of elements (atomic number, number of nucleons and atomic mass) that make up the composite. Secondly, implementing G4Material class to specify the fractional mass of each component in the composite and describe its macroscopic properties such as density. Based on the weight fraction of each compound composing the metal oxide/EPDM rubber composite listed in Table 1, the elemental weight fractions were then calculated and used to build the composite material. For example, Fig. 1 shows how the Al_2_O_3_/EPDM rubber composite was built using the G4Material class.

The PhysicsList class specifies the particle’s type and the physics processes to be used in the simulation to describe how these particles will interact with the material. In the present work, standard electromagnetic processes including photoelectric effect, Compton scattering and pair production are registered to simulate the interactions of photons with the proposed composites. In ActionInitialization class, PrimaryGeneratorAction, RunAction classes are instantiated. The PrimaryGeneratorAction class is used to control the generation of primary event and specify its type, energy, momentum and position, whereas RunAction class defines the action at the beginning and the end of each run and is responsible to record the simulated data.

**Figure 2:**
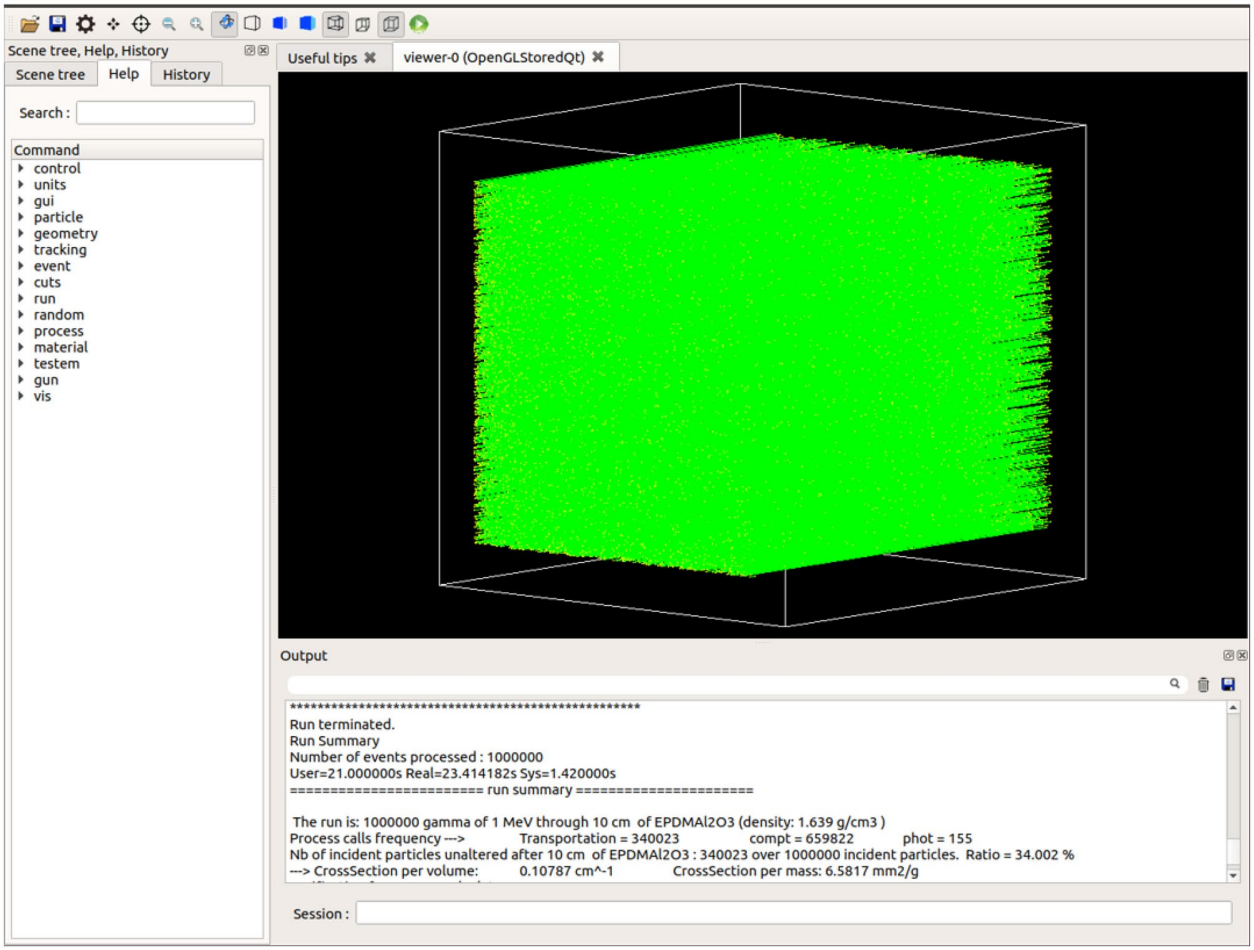
Visualization of the Geant4 simulation setup accompanied by the obtained results in the case of Al_2_O_3_/EPDM rubber composite at 1 MeV.

To obtain the linear attenuation coefficient of the present metal oxide/EPDM rubber composites, 10^6^ primary monoenergetic events are randomly shot as parallel rays at the edge of the material. This large number of incident events is used in the Monte Carlo simulation to reduce the statistical error as much as possible. The incident photon has two possibilities either transmitted or absorbed by the material. At the end of the simulation, we are able to compute the number of photons dissipated its energy by the three main interactions (photoelectric effect, Compton scattering and pair production) and those transmitted without any interaction. By applying Eq. (1), μ for each composite can be calculated, where $x=10 \mathrm{cm}$, ${I}_{0}={10}^{6}$ and *I* is the number of transmitted photons which obtained at the end of the simulation. For illustration, the Geant4 simulation setup used in the present work is visualized in Fig. 2 and accompanied by the simulation results obtained in the case of Al_2_O_3_/EPDM rubber composite at 1 MeV. Based on the simulated μ values, μ/ρ, MFP, HVL, and TVL can also be determined using Eqs. (2–5), respectively.

### NIST XCOM database

The NIST XCOM database^20^ is a web program that can generate the total attenuation coefficients as well as the partial cross sections for the photoelectric absorption, Compton scattering, and pair production processes for any element, compound, and composite at any desired photon energy. The total attenuation coefficients for mixtures are obtained as sums of the corresponding quantities for the atomic constituents. In the current study, the chemical composition of the composite with its weight fractions, as listed in Table 1, was entered into the program at an energy grid selected by the user. Then, the XCOM database printed the mass attenuation coefficients of the composite at the given photon energies. The XCOM software is utilized mainly to validate and examine the precision of the simulated results obtained by Geant4 code.

## Results and discussion

The mass attenuation coefficients (μ/ρ) for EPDM rubber composites filled with 200 phr of some metal oxides (Al_2_O_3_, CuO, CdO, Gd_2_O_3_ and Bi_2_O_3_) were simulated using Geant4 toolkit at different photon energies in the range between 0.015 and 15 MeV. To examine the precision of the simulated results^32^, the μ/ρ results simulated by the Geant4 code were compared to those calculated theoretically using the XCOM database and listed in Table 2 along with their relative deviations. The relative deviations (RD%) between Geant4 and XCOM values were computed using Eq. (13)

**Table 2:** Mass attenuation coefficient values for the EPDM composites simulated by Geant4 versus those calculated from XCOM program and their relative deviations at different photon energies.

Photon energy (MeV)	Mass attenuation coefficient (cm^2^ g^−1^)																	
	EPDM			Al_2_O_3_/EPDM			CuO/EPDM			CdO/EPDM			Gd_2_O_3_/EPDM			Bi_2_O_3_/EPDM		
	Geant4	XCOM	RD%	Geant4	XCOM	RD%	Geant4	XCOM	RD%	Geant4	XCOM	RD%	Geant4	XCOM	RD%	Geant4	XCOM	RD%
0.015	2.95396	2.98010	− 0.88	3.92251	3.93340	− 0.28	31.70681	31.64590	0.19	19.31029	19.58560	− 1.41	42.17236	42.15110	0.05	53.60809	53.41580	0.36
0.02	1.38050	1.40000	− 1.39	1.72703	1.74610	− 1.09	14.35172	14.38560	− 0.24	8.73489	8.83590	− 1.14	19.21035	19.44030	− 1.18	40.71213	40.86460	− 0.37
0.03	0.54314	0.55050	− 1.34	0.61977	0.62310	− 0.53	4.63044	4.64130	− 0.23	17.01089	16.92380	0.51	6.46256	6.47320	− 0.16	14.14609	14.16360	− 0.12
0.04	0.33331	0.33530	− 0.59	0.35345	0.35360	− 0.04	2.07451	2.09450	− 0.95	7.98204	7.97660	0.07	2.96013	2.98210	− 0.74	6.58005	6.63100	− 0.77
0.05	0.25621	0.25549	0.28	0.25797	0.25792	0.02	1.14468	1.15590	− 0.97	4.41034	4.39590	0.33	1.65330	1.65940	− 0.37	3.64992	3.68570	− 0.97
0.06	0.21847	0.21807	0.18	0.21550	0.21474	0.35	0.72971	0.73340	− 0.50	2.71002	2.70380	0.23	5.21569	5.20650	0.18	2.26809	2.29510	− 1.18
0.08	0.18404	0.18395	0.05	0.17712	0.17721	− 0.05	0.39198	0.39190	0.02	1.27197	1.27490	− 0.23	2.49581	2.49100	0.19	1.10199	1.11460	− 1.13
0.1	0.16708	0.16755	− 0.28	0.16024	0.16025	0.00	0.26625	0.26680	− 0.21	0.73370	0.73430	− 0.08	1.41112	1.41370	− 0.18	2.62609	2.63230	− 0.24
0.15	0.14564	0.14590	− 0.18	0.13860	0.13897	− 0.26	0.16653	0.16704	− 0.31	0.31059	0.31060	0.00	0.53809	0.53940	− 0.24	0.98916	0.98790	0.13
0.2	0.13166	0.13262	− 0.72	0.12616	0.12637	− 0.17	0.13564	0.13609	− 0.33	0.19622	0.19691	− 0.35	0.30067	0.30110	− 0.14	0.51667	0.51750	− 0.16
0.3	0.11515	0.11493	0.19	0.10935	0.10954	− 0.18	0.11069	0.11048	0.18	0.12758	0.12772	− 0.11	0.16237	0.16188	0.30	0.23967	0.23928	0.16
0.4	0.10325	0.10285	0.39	0.09840	0.09806	0.35	0.09742	0.09720	0.23	0.10362	0.10369	− 0.07	0.11949	0.11915	0.28	0.15701	0.15701	0.00
0.5	0.09435	0.09396	0.42	0.08970	0.08954	0.18	0.08859	0.08816	0.49	0.09111	0.09077	0.36	0.09950	0.09913	0.38	0.12123	0.12118	0.04
0.6	0.08721	0.08690	0.36	0.08305	0.08283	0.27	0.08158	0.08128	0.37	0.08261	0.08222	0.47	0.08773	0.08721	0.60	0.10182	0.10154	0.27
0.8	0.07660	0.07633	0.36	0.07297	0.07278	0.26	0.07146	0.07120	0.36	0.07125	0.07082	0.61	0.07330	0.07295	0.48	0.08051	0.08031	0.25
1	0.06884	0.06864	0.29	0.06580	0.06545	0.54	0.06421	0.06396	0.39	0.06320	0.06312	0.12	0.06433	0.06415	0.29	0.06879	0.06857	0.32
1.5	0.05594	0.05589	0.09	0.05315	0.05330	− 0.30	0.05208	0.05215	− 0.13	0.05135	0.05127	0.15	0.05171	0.05153	0.35	0.05358	0.05363	− 0.10
2	0.04809	0.04796	0.27	0.04592	0.04584	0.17	0.04519	0.04513	0.15	0.04461	0.04466	− 0.09	0.04494	0.04499	− 0.12	0.04680	0.04679	0.04
3	0.03835	0.03848	− 0.35	0.03692	0.03702	− 0.29	0.03717	0.03717	0.01	0.03766	0.03759	0.20	0.03816	0.03829	− 0.34	0.04010	0.04018	− 0.18
4	0.03282	0.03293	− 0.34	0.03198	0.03195	0.08	0.03272	0.03282	− 0.30	0.03391	0.03397	− 0.18	0.03505	0.03501	0.11	0.03714	0.03711	0.09
5	0.02925	0.02928	− 0.09	0.02857	0.02866	− 0.33	0.03007	0.03013	− 0.21	0.03197	0.03190	0.23	0.03332	0.03323	0.30	0.03546	0.03554	− 0.23
6	0.02681	0.02670	0.42	0.02624	0.02637	− 0.51	0.02829	0.02837	− 0.28	0.03059	0.03063	− 0.12	0.03220	0.03221	− 0.05	0.03477	0.03474	0.10
8	0.02336	0.02332	0.17	0.02340	0.02344	− 0.20	0.02625	0.02629	− 0.15	0.02930	0.02938	− 0.27	0.03129	0.03135	− 0.20	0.03416	0.03429	− 0.39
10	0.02129	0.02123	0.26	0.02170	0.02168	0.11	0.02524	0.02522	0.11	0.02897	0.02898	− 0.03	0.03120	0.03128	− 0.26	0.03468	0.03457	0.33
15	0.01841	0.01842	− 0.04	0.01949	0.01944	0.27	0.02425	0.02422	0.12	0.02915	0.02928	− 0.45	0.03228	0.03226	0.05	0.03637	0.03630	0.19

13 $$RD\%=\frac{{(\mu /\rho )}_{Geant4}-{(\mu /\rho )}_{XCOM}}{{(\mu /\rho )}_{XCOM}}$$

**Figure 3:**
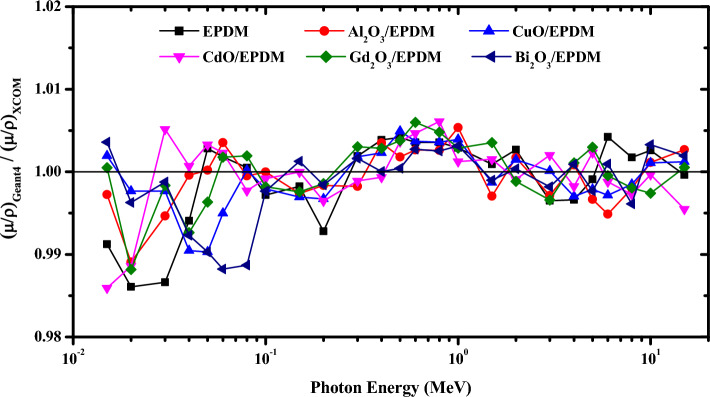
Ratios of μ/ρ values simulated by Geant4 toolkit to those obtained from XCOM program.

As can be remarked from Table 2, the relative deviations vary in the range of − 1.39 to 0.42% for EPDM, − 1.09 to 0.54 for Al_2_O_3_/EPDM, − 0.97 to 0.49% for CuO/EPDM, − 1.41 to 0.61% for CdO/EPDM, − 1.18 to 0.6% for Gd_2_O_3_/EPDM and − 1.18 to 0.36% for Bi_2_O_3_/EPDM rubber composites. Therefore, Table 2 confirms that the Geant4 simulated results of μ/ρ for all the investigated metal oxides/EPDM rubber composites are fairly match with those obtained theoretically from XCOM database through all energy regions which insure the validity of our Geant4 simulation code. Furthermore, in order to clarify the discrepancy between the simulated results and the theoretically calculated results, Fig. 3 depicts the ratios of μ/ρ values simulated by Geant4 toolkit to those obtained from XCOM program. It is clear from Fig. 3 that, the gaps between Geant4 and XCOM results are very small which can be ascribed to the high accuracy of the geometry construction and the electromagnetic physics models employed by Geant4 Monte Carlo toolkit.

**Figure 4:**
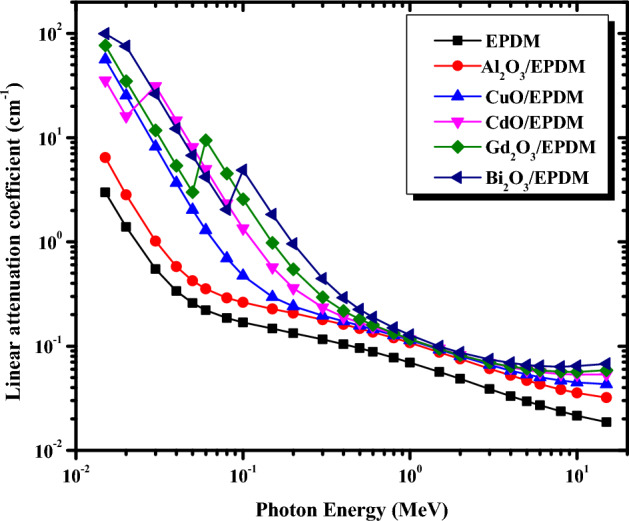
Geant4 linear attenuation coefficients of the selected metal oxide/EPDM rubber composites as a function of photon energy.

The linear attenuation coefficient (μ) is a fundamental shielding parameter that can be used to assist the effect of adding different metal oxides on the gamma-ray protective capability of the EPDM rubber matrix. The simulated data of μ for the current metal oxide/EPDM rubber composites versus incident photon energy in the range between 0.015 and 15 MeV is plotted in Fig. 4. It evident from Fig. 4 that, μ values for all the proposed EPDM rubber composites are dependent on the energy of the incident gamma-ray and the type of the added metal oxide. Figure 4 demonstrates that adding 200 phr of different metal oxides to the EPDM rubber matrix increases the linear attenuation coefficients, and the enhancement is more significant at low photon energies between (0.015 < E < 0.3 MeV). On the other hand, increasing the photon energy in this region, sharply decreases μ values. This is due to the fact that the cross-sections for photoelectric interactions are sufficiently high in this energy range, and photons are mostly to be absorbed primarily by the photoelectric effect, which depends on Z^4^/E^3.5^^33^, where Z is the atomic number of the absorbing element and E is the incident gamma-ray energy. This illustrates why the highest μ values were found for Bi_2_O_3_/EPDM (z = 83 for Bi), while the lowest μ values were obtained for Al_2_O_3_/EPDM (z = 13 for Al). As an exception to this trend, there are three sudden peaks in μ values occurring at 0.0267 MeV for CdO/EPDM, 0.0502 MeV for Gd_2_O_3_/EPDM, and 0.0905 MeV for Bi_2_O_3_/EPDM composites. These sharp peaks are due to the K absorption edges of Cd, Gd, and Bi, respectively. It is also noticed that the composite CuO/EPDM shows discrepancies at low photon energies, where CuO/EPDM composite has greater μ values compared to CdO/EPDM. This discrepancy occurred because Cu has a K absorption edge at 0.0089 MeV. Therefore, at low energies of 0.015 and 0.02 MeV, CuO/EPDM has effective shielding properties compared to CdO/EPDM.

As the photon energy increases further between (0.3 < E < 2 MeV), the μ values slowly decrease. Meanwhile, the type of added metal oxide has no remarkable effect on the μ values at the same energy. That is to say, each metal oxide/EPDM rubber composite approximately has the same value of μ over this energy range. This is due to the impact of photoelectric absorption diminishing at this intermediate energy range and Compton scattering becoming the predominant mechanism. In fact, the Compton scattering relies on the number of electrons per unit mass, which is proportional to Z/M^34^ (where Z and M are the atomic and mass numbers, respectively). This ratio is roughly equal to 0.5 for most elements. In other words, at photon energies where Compton effect dominates, μ values tend to be very close for all elements. Consequently, changing the type of filler in the EPDM rubber matrix in this energy range does not have a significant effect on the μ values. However, at higher energies greater than 2 MeV, the difference between μ values becomes comparatively wider and decreases gradually as the energy of the incident photons increases due to the pair production process, which has a cross-section proportional to log E^35^.

**Figure 5:**
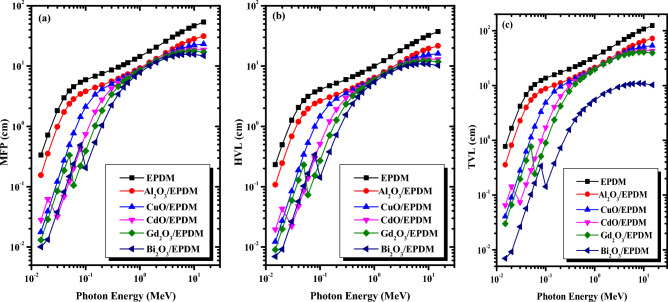
The variation of the (**a**) mean free path, (**b**) half value layer and (**c**) tenth value layer with photon energy for the selected metal oxide/EPDM rubber composites.

MFP, HVL, and TVL, commonly calculated shielding parameters, are used to evaluate the effectiveness of the shielding materials. MFP represents the distance between two successive collisions inside the shield; hence a lower MFP implies a greater number of interactions and more significant attenuation. At the same time, HVL and TVL represent the absorber thicknesses required to reduce the incident gamma-ray intensity to 50% and 10% of its initial value, respectively. The better shielding materials have low MFP, HVL, and TVL values. The variation of the MFB, HVL, and TVL values for the present metal oxide/EPDM rubber composites versus incident photon energy is depicted in Fig. 5a–c, respectively. As can be seen from Fig. 5, the variations of MFP, HVL, and TVL with photon energy apparently have the same trend, but the difference is in magnitude. The MFB, HVL, and TVL values for all the selected samples tend to increase with increasing the photon energy while decreasing according to the type of the added metal oxide.

EPDM has the highest MFP values ranging from 0.34 to 53.77 cm, while Bi_2_O_3_/EPDM rubber composite has the lowest MFP values ranging from 0.01 to 14.82 cm at 0.015 MeV and 15 MeV, respectively. Figure 5 reveals that the MFP, HVL and TVL are decreasing in the order of EPDM < Al_2_O_3_/EPDM < CuO/EPDM < CdO/EPDM < Gd_2_O_3_/EPDM < Bi_2_O_3_/EPDM at the same photon energy, with some exceptions, there are sudden decline in the MFP, HVL and TVL values occurring at 0.0267 MeV for CdO/EPDM, 0.0502 MeV for Gd_2_O_3_/EPDM, and 0.0905 MeV for Bi_2_O_3_/EPDM composites. These sharp drops are due to the K absorption edges of Cd, Gd, and Bi, respectively, consistent with the former results of the linear attenuation coefficients. Consequently, incorporating metal oxides into the EPDM rubber matrix reduces the MFP, HVL, and TVL values leading to an improvement in the shielding effectiveness of the EPDM rubber composites.

**Figure 6:**
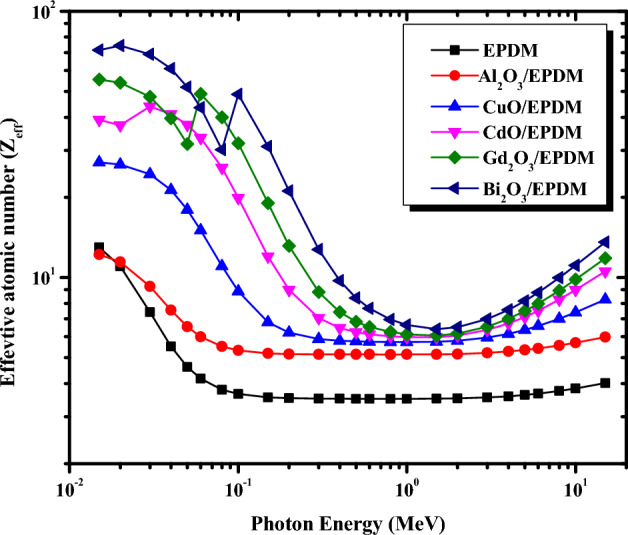
Variations of the effective atomic number for the metal oxide/EPDM rubber composites with photon energy.

**Figure 7:**
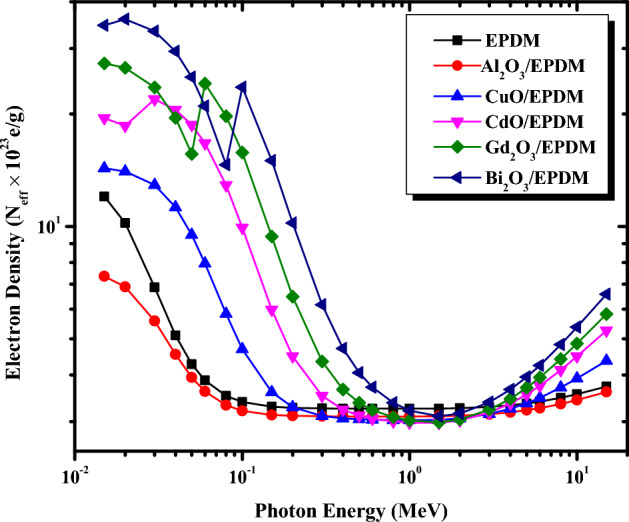
Variations of the effective electron density for the metal oxide/EPDM rubber composites with photon energy.

The effective atomic number Z_eff_ and the electron density N_eff_ for the metal oxide/EPDM rubber composites were computed theoretically in the energy range between 0.015 and 15 MeV and depicted in Figs. 6 and 7, respectively. Z_eff_ and N_eff_ were determined using the mass attenuation coefficient obtained from the XCOM database for each constituent element in the composite sample as described by Eqs.(6) and (7). It is evident from Figs. 6 and 7 that Z_eff_ and N_eff_ depend on the incident photon energy and the type of the added metal oxide. Figure 6 demonstrates that in the energy range between 0.015 and 0.2 MeV, Z_eff_ for all the metal oxide/EPDM rubber composites falls rapidly with the increase in the photon energy since the photoelectric effect is the predominant interaction in this energy range which varies inversely with E^3.5^. Further increase in the photon energy between 0.3 and 3 MeV, the Z_eff_ values are approximately constant for each composite due to the Compton scattering cross-section in this energy range. For the high energy region between 3 and 15 MeV, a slow increase in the Z_eff_ values is observed by increasing the photon energy due to the predominance of the pair production. It is also clear from Fig. 6 that Z_eff_ values increase apparently in the order of EPDM < Al_2_O_3_/EPDM < CuO/EPDM < CdO/EPDM < Gd_2_O_3_/EPDM < Bi_2_O_3_/EPDM at the same photon energy. The highest Z_eff_ is obtained for Bi_2_O_3_/EPDM composite and ranges from 71.42 to 13.60. In contrast, the lowest Z_eff_ is obtained for EPDM and ranges from 12.97 to 4.01 in the energy range between 0.015 and 15 MeV, respectively. Figure 7 shows N_eff_ is also a function in the incident gamma-ray energy. The dependence of N_eff_ on the incident photon energy and the type of incorporated metal oxide can be discussed as in the Z_eff_ section.

**Figure 8:**
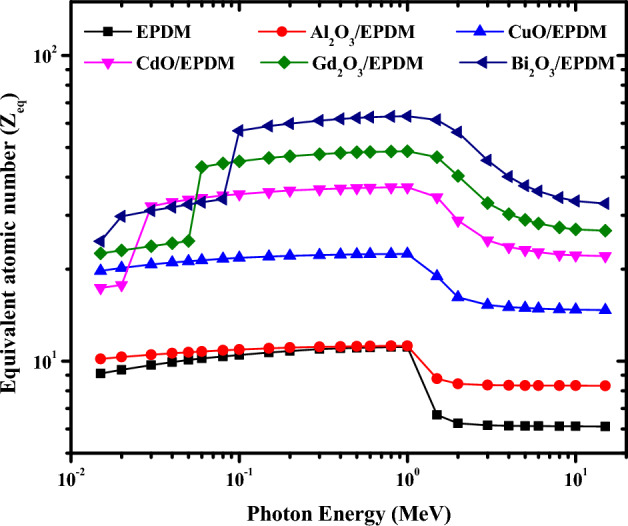
Variations of the equivalent atomic number for the metal oxide/EPDM rubber composites with photon energy.

The interactions of gamma radiation at given energy with a material depend on the atomic number of the interacting medium. For this purpose, it is crucial to calculate the composite's equivalent atomic number (Z_eq_), which is synonymous with the elemental atomic number. The composite material with the higher equivalent atomic number is the best protective material. Figure 8 shows the variation of Z_eq_ values for the metal oxide/EPDM rubber composites against gamma-ray energy. It is obvious that the Z_eq_ increases gradually to reach its maximum value for all the composites at 1 MeV due to the Compton scattering process. Then, it decreases rapidly when the gamma-ray energy exceeds 1 MeV due to the pair production process. Figure 8 shows that the insertion of metal oxides into the EPDM matrix causes the Z_eq_ to increase at the same gamma-ray energy. The highest Zeq was found for the Bi_2_O_3_/EPDM composite, while the lowest Z_eq_ was for the EPDM composite. Furthermore, due to the K absorption edges of Cd, Gd, and Bi, there are three sudden peaks in Z_eq_ values occurring at 0.0267 MeV for CdO/EPDM, 0.0502 MeV for Gd_2_O_3_/EPDM, and 0.0905 MeV for Bi_2_O_3_/EPDM composites, respectively. Among all the studied composites, Bi_2_O_3_/EPDM composite has better shielding ability than other EPDM composites, which is in line with the former results.

**Figure 9:**
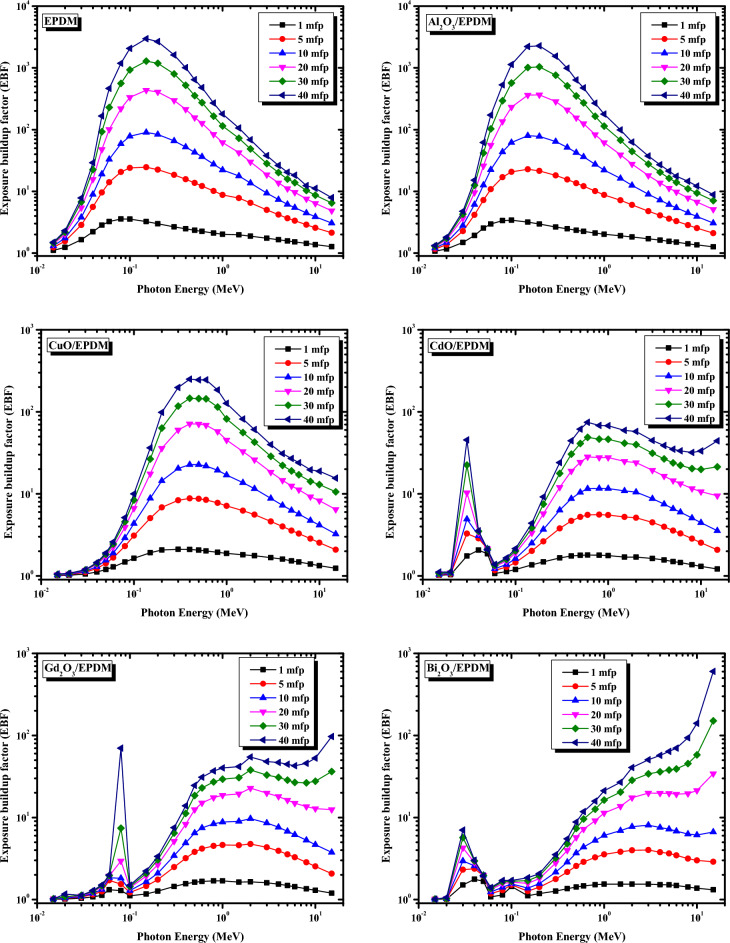
Variations of the exposure buildup factor with gamma ray energy for the metal oxide/EPDM rubber composites at various mfps.

In designing and developing an efficient shielding material, the exposure buildup factor (EBF) must be considered to examine the effects of multiple gamma-ray scattering. EBF, always greater than 1, corrects Lambert–Beer's equation due to the contribution of multiple photon interactions caused by the secondary gamma-ray emissions. Figure 9 depicts the variation of the EBF versus photon energy between 0.015 and 15 MeV for the metal oxide/EPDM rubber composites at penetration depths 1, 5, 10, 20, 30 and 40 mfp. As can be seen from Fig. 9, the EBF values in case of EPDM, Al_2_O_3_/EPDM, CuO/EPDM rubber composites showing apparently the same trend against the incident photon energies. In this trend, the EBF values are much higher at moderate gamma-ray energies between 0.08 and 0.5 MeV, where the Compton scattering process generates secondary photons and these photons are not totally removed. Still, they are prone to multiple scattering leading to a remarkable rise in the EBF values. On the other hand, at low and high gamma-ray energies, the EBF values are much smaller compared to moderate energies. This trend is due to the predominance of the photoelectric effect and pair production mechanisms, respectively, in which the photons are entirely absorbed or severely depleted their energies in low and high-energy regions. As an exception to the previous trend, in case of CdO/EPDM, Gd_2_O_3_/EPDM, and Bi_2_O_3_/EPDM, the EBF values showing sudden peaks at low energies. These sharp peaks are due to the K absorption edges of Cd, Gd, and Bi, respectively. Moreover, it is also evident from Fig. 9 that the EBF values for all the metal oxide/EPDM rubber composites increase by increasing the penetration depths from 1 to 40 mfp. This behavior can be attributed to the generation of multiple photons due to increased interactions of photons at large penetration depths.

**Figure 10:**
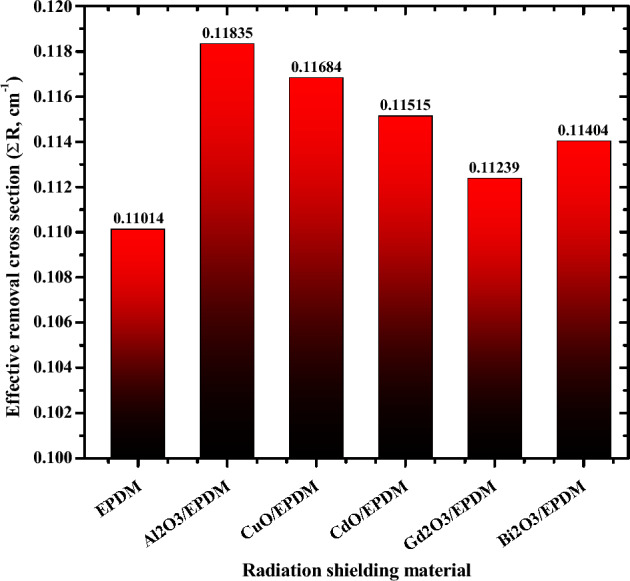
Effective removal cross section of fast neutrons for the selected metal oxide/EPDM rubber composites.

Finally, the macroscopic effective removal cross section for fast neutrons (Ʃ_R_) was evaluated for the investigated metal oxide/EPDM rubber samples using MRCsC software^36^. MRCsC is a user-friendly software developed to accurately predict macroscopic effective removal cross-section, Σ_R_, (in cm^−1^) of fast neutrons for different shielding composites. It is a simple interface designed with minimum input requirements from the user. By inserting the material density and the corresponding element's weight fraction, the user can determine the macroscopic effective removal cross-section. Figure 10 shows how adding Al_2_O_3_, CuO, CdO, Gd_2_O_3_ and Bi_2_O_3_ to EPDM rubber improves its ability to attenuate fast neutrons. Also the Ʃ_R_ values of the EPDM rubber containing Aluminum, copper, cadmium, gadolinium and bismuth oxides are increasing in the order of EPDM (0.11014 cm^−1^) < Gd_2_O_3_/EPDM (0.11239 cm^−1^) < Bi_2_O_3_/EPDM (0.11404 cm^−1^) < CdO/EPDM (0.11515 cm^−1^) < CuO/EPDM (0.11684 cm^−1^) < Al_2_O_3_/EPDM (0.11835 cm^−1^).

## Conclusions

The current work investigates the radiation shielding features of EPDM rubber composites filled with 200 phr of different metal oxides (Al_2_O_3_, CuO, CdO, Gd_2_O_3_, and Bi_2_O_3_) using the Geant4 Monte Carlo simulation toolkit. From this study, it can be concluded that the Geant4 simulation software offers accurate and flexible use to predict the shielding capability of the tested materials. The study demonstrates that the gamma-radiation shielding performance of the proposed metal oxide/EPDM rubber composites is increasing in the order of EPDM < Al_2_O_3_/EPDM < CuO/EPDM < CdO/EPDM < Gd_2_O_3_/EPDM < Bi_2_O_3_/EPDM. Furthermore, three sudden increases in the shielding capability in some composites occur at 0.0267 MeV for CdO/EPDM, 0.0502 MeV for Gd_2_O_3_/EPDM, and 0.0905 MeV for Bi_2_O_3_/EPDM composites. This increase in the shielding performance is due to the K absorption edges of Cd, Gd, and Bi, respectively. Moreover, the macroscopic effective removal cross-section for fast neutrons (Ʃ_R_) was also evaluated for the current composites. Ʃ_R_ varies between 0.11014 cm^−1^ for EPDM to 0.11835 cm^−1^ for Al_2_O_3_/EPDM. This theoretical investigation explored the potential use of the proposed metal oxide/EPDM rubber composites as radiation protective materials for workers in radiation facilities to be used as clothing and gloves.

## Data Availability

The datasets used and/or analyzed during the current study available from the corresponding author on reasonable request.
